# The 100 most cited articles in ectopic pregnancy: a bibliometric analysis

**DOI:** 10.1186/s40064-016-3503-8

**Published:** 2016-10-19

**Authors:** Xue-lian Chen, Zi-ru Chen, Zhen-lan Cao, Ke Han, Ya-wen Tong, Xiao-hui Xiang, Chun-xiu Hu

**Affiliations:** 1 Department of Obstetrics and Gynecology, Affiliated Hospital, Logistics University of the Chinese People’s Armed Police Force, 220 Chenglin Road, Hedong District, Tianjin, 300162 China; 2 Department of Equipment, Affiliated Hospital, Logistics University of the Chinese People’s Armed Police Force, Tianjin, 300162 China; 3 Department of Hepatopancreatobiliary and Splenic Medicine, Affiliated Hospital, Logistics University of the Chinese People’s Armed Police Force, 220 Chenglin Road, Hedong District, Tianjin, 300162 China

**Keywords:** Citation analysis, Bibliometrics, Ectopic pregnancy

## Abstract

Ectopic pregnancy (EP) remains a major gynecological emergency and is a cause of morbidity or even mortality in women. As a consequence, top citation analysis of EP research in database of the Science Citation Index Expanded is needed to assess the publication trends of leading countries/territories and institutes as well as the research hotspots of EP. A total of 4881 articles relevant to EP were retrieved in the database of the Science Citation Index Expanded from 1965 to present, in which the 100 top-cited articles were selected for further analysis. The number of citations ranged from 81 to 482 (131.57 ± 69.76), with a time span of 40 years between 1969 and 2009. These citation classics came from 14 countries, and 65 of the articles came from the United States. Yale University in Connecticut led the list of classics with six papers. The 100 top-cited articles were published in 32 journals, in which the journal of Fertility and Sterility published the most (23 papers). Stovall TG and Ling FW published the highest number of studies (6 papers each). Articles that originated in the United States and that were published in high-impact journals were most likely to be cited in the field of EP research. Bibliometric analysis was used to provide a historical perspective on the progress in EP research over the past 50 years. Citation analysis is a feasible tool to comprehensively recognize the advances of EP research in the past and future research.

## Background

Ectopic pregnancy (EP), which is the implantation of a fertilized ovum outside the endometrial cavity and has an approximate incidence of 1.5–2.0 % in all pregnancies, is a potentially life-threatening disease (Chang et al. 2003). With increasing in vitro fertilization (IVF) procedures, there is an increase of Eps (Ramer et al. 2016; Sisti et al. 2016). Currently, it remains a major problem in contemporary gynecological practice and continues to be an important cause of morbidity and mortality in women. EP is also a clinical manifestation of poor fertility prognosis and adverse outcomes in subsequent pregnancies (Musa et al. 2009). Although oviduct inflammation, the history of tubal pregnancy and tubal surgery, the application of assisted reproductive technology (ART) and so on are known as the pathogenesis of EP by now. However, the definite mechanism of it is still missing. Therefore, many specialists and researchers have focused their efforts on EP to gain a better understanding of the mechanism of this disease and develop new methods for the diagnosis and treatment of this issue.

Citation is an author’s reference to a previous work that acknowledges the relevance of that work in contributing to the completion of the author’s current paper (Kavanagh et al. 2013). While number of citations is not the only factor in determining an article’s relevance, it is arguably our best marker for articles that have been influential in the field (Baldwin et al. 2013). Citation analysis involves ranking and evaluating an article or journal based on the frequency of citation that it receives (Murray et al. 2012). The frequency of citation has significant implications for authors, journals, institutions and even nations (Moed 2009). Citation analysis is the bibliometric process that is used to examine the citation history of a particular paper by examining the citations attributed to that publication. Usually, it involves ranking and evaluating an article or journal based on the number of citations that it has received.

To systematically review the citation classics dedicated to EP, we evaluated the current literature for the 100 most frequently cited articles in an attempt to provide a bibliometric perspective of the progress in this field. To our knowledge, this is the first study to quantify and analyze the most frequently cited papers to review the history of EP.

## Methods

The database (Web of Science Expanded citation index) of the Institute for Scientific Information (ISI) from 1965 to 2015 was searched using the keyword “ectopic pregnanc*” to identify the citation classics. This database includes peer-reviewed publications indexed from more than 10,000 high-impact journals worldwide. Only papers that had been published as an “article” were selected for further study and no language restriction criteria was applied in our study. Each article in the top 100 cited list was reviewed, and the information including number of citations, authorship, journals, institution and country of origin, and year of publication was retrieved. Country of origin was defined based on the first author’s address. The addresses of other authors were noted to determine whether international collaboration was involved.

## Results

**Fig. 1:**
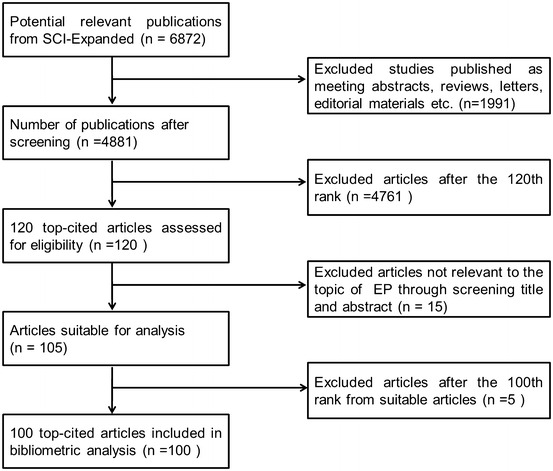
Flowchart of the selection process for the 100 top-cited articles in ectopic pregnancy

The 100 most frequently cited articles related to EP were identified using Science Citation Index Expanded (SCI-expanded). A total of 6872 papers were identified in the period from 1965 to 2015, with 4881 classified as “article”, 384 classified as “review”, 499 classified as “letter”, 245 classified as “proceeding paper”, 410 classified as “meeting abstract”; the remaining were classified as “other”. The selection process of the articles is shown in Fig. 1, and the top 100 cited articles composing our final list are shown in Table 1. By reading the abstract or full text of the original 100 most cited articles, a total of 15 articles like the 15th paper written by Gaydos et al. (1998) and the 25th one contributed by Conway et al. (1997) were eliminated because of its minor relevance to EP.

**Table 1:** List of the 100 top-cited articles in ectopic pregnancy

Rank	Articles	Citation times
1	Kalman S, Mitchell W, Marathe R, et al.: Comparative genomes of *Chlamydia pneumoniae* and *C. trachomatis*. Nat Genet. 1999. 21(4):385–389	482
2	Westroml L, Joesoef R, Reynolds G, et al.: Pelvic inflammatory disease and fertility—a cohort study of 1844 women with laparoscopically verified disease and 657 control women with normal laparoscopic results. Sex Transm Dis. 1992. 19(4):185–192	384
3	Cates W, Wasserheit JN: Genital chlamydial infections—epidemiology and reproductive sequelae. Am J Obstet Gynecol.1991. 164(6):1771–1781	359
4	Miller WC, Ford CA, Morris M, et al.: Prevalence of chlamydial and gonococcal infections among young adults in the United States. JAMA—J Am Med Assoc. 2004. 291(18):2229–2236	321
5	Andersen AMN, Wohlfahrt J, Christens P, et al.: Maternal age and fetal loss: population based register linkage study. Brit Med J. 2000. 320(7251):1708–1712	317
6	Stovall TG, Ling FW: Single-dose methotrexate—an expanded clinical-trial. Am J Obstet Gynecol. 1993. 168(6):1759–1765	251
7	Kadar N, Caldwell BV, Romero R: A method of screening for ectopic pregnancy and its indications. Obstet Gynecol. 1981. 58(2):162–166	242
8	Kadar N, Devore G, Romero R: Discriminatory Hcg zone—its use in the sonographic evaluation for ectopic pregnancy. Obstet Gynecol. 1981. 58(2):156–161	235
9	Pouly JL, Mahnes H, Mage G, et al.: Conservative laparoscopic treatment of 321 ectopic pregnancies. Fertil Steril. 1986. 46(6):1093–1097	230
10	Peyron R, Aubeny E, Targosz V, et al.: Early termination of pregnancy with mifepristone (Ru-486) and the orally active prostaglandin misoprostol. New Engl J Med. 1993. 328(21):1509–1513	224
11	Westrom L, Bengtsson LP, Mardh PA: Incidence, trends, and risks of ectopic pregnancy in a population of women. Brit Med J. 1981. 282(6257):15–18	219
12	Lundorff P, Hahlin M, Kallfelt B, et al.: Adhesion formation after laparoscopic surgery in tubal pregnancy—a randomized trial versus laparotomy. Fertil Steril. 1991. 55(5):911–915	201
13	Breen JL: A 21 year survey of 654 ectopic pregnancies. Am J Obstet Gynecol. 1970. 106(7):1004	197
14	Jurkovic D, Hillaby K, Woelfer B, et al.: First-trimester diagnosis and management of pregnancies implanted into the lower uterine segment cesarean section scar. Ultrasound Obst Gynecol. 2003. 21(3):220–227	182
15	Stovall TG, Ling FW, Gray LA: Single-dose methotrexate for treatment of ectopic pregnancy. Obstet Gynecol. 1991. 77(5):754–757	172
16	Bruhat MA, Manhes H, Mage G, et al.: Treatment of ectopic pregnancy by means of laparoscopy. Fertil Steril. 1980. 33(4):411–414	169
17	Castles A, Adams EK, Melvin CL, et al.: Effects of smoking during pregnancy—five meta-analyses. Am Journal Prev Med. 1999. 16(3):208–215	167
18	Stovall TG, Ling FW, Gray LA, et al.: Methotrexate treatment of unruptured ectopic pregnancy—a report of 100 cases. Obstet Gynecol. 1991. 77(5):749–753	162
19	Peipert JF: Genital chlamydial infections. New Engl J Med. 2003. 349(25):2424–2430	156
20	Balen AH, Tan SL, Macdougall J, et al.: Miscarriage rates following invitro fertilization are increased in women with polycystic ovaries and reduced by pituitary desensitization with buserelin. Hum Reprod. 1993. 8(6):959–964	156
21	Beral V: Epidemiological study of recent trends in ectopic pregnancy. Brit J Obstet Gynaec. 1975. 82(10):775–782	155
22	Ory SJ, Villanueva AL, Sand PK, et al.: Conservative treatment of ectopic pregnancy with methotrexate. Am J Obstet Gynecol. 1986. 154(6):1299–1306	152
23	Hillis SD, Owens LM, Marchbanks PA, et al.: Recurrent chlamydial infections increase the risks of hospitalization for ectopic pregnancy and pelvic inflammatory disease. Am J Obstet Gynecol. 1997. 176(1):103–107	151
24	Hillis SD, Joesoef R, Marchbanks PA, et al.: Delayed care of pelvic inflammatory disease as a risk factor for impaired fertility. Am J Obstet Gynecol. 1993. 168(5):1503–1509	148
25	Seow KM, Huang LW, Lin YH, et al.: Cesarean scar pregnancy: issues in management. Ultrasound Obst Gyn. 2004. 23(3):L247–253	143
26	Ankum WM, Mol BWJ, Vander Veen F, et al.: Risk factors for ectopic pregnancy: a meta-analysis. Fertil Steril. 1996. 65(6):1093–1099	142
27	Lapensee L, Paquette Y, Bleau G: Allelic polymorphism and chromosomal localization of the human oviductin gene (Muc9). Fertil Steril. 1997. 68(4):702–708	137
28	Chow JM, Yonekura ML, Richwald GA, et al.: The association between *Chlamydia trachomatis* and ectopic pregnancy—a matched-pair, case–control study. JAMA—J Am Med Assoc. 1990. 263(23):3164–3167	137
29	Ness RB, Soper DE, Holley RL, et al.: Effectiveness of inpatient and outpatient treatment strategies for women with pelvic inflammatory disease: results from the pelvic inflammatory disease evaluation and clinical health (PEACH) randomized trial. Am J Obstet Gynecol. 2002. 186(5):929–937	132
30	Hemminki E, Merilainen J: Long-term effects of cesarean sections: ectopic pregnancies and placental problems. Am J Obstet Gynecol. 1996. 174(5):1569–1574	132
31	Lipscomb GH, McCord ML, Stovall TG, et al.: Predictors of success of methotrexate treatment in women with tubal ectopic pregnancies. New Engl J Med. 1999. 341(26):1974–1978	130
32	Egger M, Low N, Smith GD, et al.: Screening for chlamydial infections and the risk of ectopic pregnancy in a county in Sweden: ecological analysis. Brit Med J. 1998. 316(7147):1776–1780	130
33	Cacciatore B. Stenman UH. Ylostalo P: Diagnosis of ectopic pregnancy by vaginal ultrasonography in combination with a discriminatory serum Hcg level of 1000-Iu/1 (Irp). Brit J Obstet Gynaec. 1990. 97(10):904–908	129
34	Sauer MV, Gorrill MJ, Rodi IA, et al.: Nonsurgical management of unruptured ectopic pregnancy—an extended clinical-trial. Fertil Steril. 1987. 48(5):752–755	129
35	Wald NJ, Hackshaw AK: Cigarette smoking: an epidemiological overview. Brit Med Bull. 1996. 52(1):3–11	126
36	Decherney A, Kase N: Conservative surgical management of unruptured ectopic pregnancy. Obstet Gynecol. 1979. 54(4):451–455	124
37	Whittington WLH, Kent C, Kissinger P, et al.: Determinants of persistent and recurrent *Chlamydia trachomatis* infection in young women—results of a multicenter cohort study. Sex Transm Dis. 2001. 28(2):117–123	123
38	Craven CM, Morgan T, Ward K: Decidual spiral artery remodelling begins before cellular interaction with cytotrophoblasts. Placenta. 1998. 19(4):241–252	122
39	Stovall TG, Ling FW, Buster JE: Outpatient chemotherapy of unruptured ectopic pregnancy. Fertil Steril. 1989. 51(3):435–438	121
40	Lau S, Tulandi T: Conservative medical and surgical management of interstitial ectopic pregnancy. Fertil Steril. 1999. 72(2):207–215	117
41	Schoolcraft WB, Surrey ES, Gardner DK: Embryo transfer: techniques and variables affecting success. Fertil Steril. 2001. 76(5):863–870	115
42	Rubin GL, Peterson HB, Dorfman SF, et al.: Ectopic pregnancy in the United-States—1970 through 1978. JAMA-J Am Med Assoc. 1983. 249(13):1725–1729	115
43	Ostergaard L, andersen B, Moller JK, et al.: Home sampling versus conventional swab sampling for screening of *Chlamydia trachomatis* in women: a cluster-randomized 1-year follow-up study. Clin Infect Dis. 2000. 31(4):951–957	113
44	Bouyer J, Coste J, Shojaei T, et al.: Risk factors for ectopic pregnancy: a comprehensive analysis based on a large case–control, population-based study in France. Am J Epidemiol. 2003. 157(3):185–194	112
45	Diav-Citrin O, Park YH, Veerasuntharam G, et al.: The safety of mesalamine in human pregnancy: a prospective controlled cohort study. Gastroenterology. 1998. 114(1):23–28	112
46	Godin PA, Bassil S, Donnez J: An ectopic pregnancy developing in a previous caesarian section scar. Fertil Steril. 1997. 67(2):398–400	111
47	Rogers JM: Tobacco and pregnancy. Reprod Toxicol. 2009. 28(2):152–160	107
48	Rein DB, Kassler WJ, Irwin KL, et al.: Direct medical cost of pelvic inflammatory disease and its sequelae: decreasing, but still substantial. Obstet Gynecol. 2000. 95(3):397–402	107
49	Barnhart K, Mennuti MT, Benjamin I, et al.: Prompt diagnosis of ectopic pregnancy in an emergency department setting. Obstet Gynecol. 1994. 84(6);1010–1015	106
50	Coulam CB: Epidemiology of recurrent spontaneous-abortion. Am J Reprod Immunol. 1991. 26(1):23–27	106
51	Decherney AH, Diamond MP: Laparoscopic salpingostomy for ectopic pregnancy. Obstet Gynecol. 1987. 70(6):948–950	106
52	Hausknecht RU: Methotrexate and misoprostol to terminate early-pregnancy. New Engl J Med. 1995. 333(9):537–540	105
53	Ory HW: Ectopic pregnancy and intrauterine contraceptive devices—new perspectives. Obstet Gynecol.1981. 57(2):137–144	104
54	Timortritsch IE, Monteagudo A, Matera C, et al.: Sonographic evolution of cornual pregnancies treated without surgery. Obstet Gynecol. 1992. 79(6):1044–1049	103
55	Fernandez H, Rainhorn JD, Papiernik E, Bellet D, Frydman R: Spontaneous resolution of ectopic pregnancy. Obstet Gynecol. 1988. 71(2):171–174	103
56	Asplin BR, Rhodes KV, Levy H, et al.: Insurance status and access to urgent ambulatory care follow-up appointments. JAMA—J Am Med Assoc. 2005. 294(10):1248–1254	101
57	Critchley HOD, Jones RL, Lea RG, et al.: Role of inflammatory mediators in human endometrium during progesterone withdrawal and early pregnancy. J Clin Endocr Metab. 1999. 84(1):240–248	101
58	Frates MC, Benson CB, Doubilet PM, et al.: Cervical ectopic pregnancy—results of conservative treatment. Radiology. 1994. 191(3):773–775	100
59	Marchbanks PA, Annegers JF, Coulam CB, et al.: Risk-factors for ectopic pregnancy—a population-based study. JAMA-J Am Med Assoc. 1988. 259(12):1823–1827	99
60	Bouyer J, Coste J, Fernandez H, et al.: Sites of ectopic pregnancy: a 10 year population-based study of 1800 cases. Hum Reprod. 2002. 17(12):3224–3230	98
61	Brunham RC, Binns B, Mcdowell J, et al.: *Chlamydia trachomatis* infection in women with ectopic pregnancy. Obstet Gynecol. 1986. 67(5):722–726	97
62	Kobayash. M, Hellman LM, Fillisti LP: Ultrasound—an aid in diagnosis of ectopic pregnancy. Am J Obstet Gynecol. 1969. 103(8):1131	96
63	Pisarska MD, Carson SA, Buster JE: Ectopic pregnancy. Lancet. 1998. 351(9109):1115–1120	95
64	Bradley WG, Fiske CE, Filly RA: The double sac sign of early intrauterine pregnancy—use in exclusion of ectopic pregnancy. Radiology. 1982. 143(1):223–226	95
65	Glock JL, Johnson JV, Brumsted JR: Efficacy and safety of single-dose systemic methotrexate in the treatment of ectopic pregnancy. Fertil Steril. 1994. 62(4):716–721	94
66	Brown DL, Doubilet PM: Transvaginal sonography for diagnosing ectopic pregnancy—positivity criteria and performance-characteristics. J Ultras Med. 1994. 13(4):259–266	94
67	Schwartz RO, Dipietro DL: Beta-HCG as a diagnostic-aid for suspected ectopic pregnancy. Obstet Gynecol. 1980. 56(2):197–203	94
68	Garcia AJ, Aubert JM, Sama J, et al.: Expectant management of presumed ectopic pregnancies. Fertil Steril. 1987. 48(3):395–400	93
69	Aral SO, Mosher WD, Cates W: Vaginal douching among women of reproductive age in the United-States—1988. Am J Public Health. 1992. 82(2):210–214	92
70	Fleischer AC, Pennell RG, Mckee MS, et al.: Ectopic pregnancy—features at transvaginal sonography. Radiology. 1990. 174(2):375–378	92
71	Logerotlebrun H, Demouzon J, Bachelot A, et al.: Pregnancies and births resulting from in-vitro fertilization—French National Registry, analysis of data 1986–1990. Fertil Steril. 1995. 64(4):746–756	91
72	Marcus SF, Brinsden PR: Analysis of the incidence and risk-factors associated with ectopic pregnancy following in-vitro fertilization and embryo-transfer. Hum Reprod. 1995. 10(1):199–203	91
73	Brumsted J, Kessler C, Gibson C, et al.: A comparison of laparoscopy and laparotomy for the treatment of ectopic pregnancy. Obstet Gynecol. 1988. 71(6):889–892	91
74	Romero R, Kadar N, Jeanty P, et al.: Diagnosis of ectopic pregnancy—value of the discriminatory human chorionic-gonadotropin zone. Obstet Gynecol. 1985. 66(3):357–360	91
75	Decherney AH, Maheaux R, Naftolin F: Salpingostomy for ectopic pregnancy in the sole patent oviduct—reproductive outcome. Fertil Steril. 1982. 37(5):619–622	91
76	Barnhart KT: Ectopic pregnancy. New Engl J Med. 2009. 361(4):379–387	90
77	Brenner PF, Roy S, Mishell DR: Ectopic pregnancy—study of 300 consecutive surgically treated cases. JAMA-J Am Med Assoc. 1980. 243(7):673–676	90
78	Marks WM, Filly RA, Callen PW, et al.: Decidual cast of ectopic pregnancy—confusing ultrasonographic appearance. Radiology. 1979. 133(2):451–454	90
79	Kaplan BC, Dart RG, Moskos M, et al.: Ectopic pregnancy: prospective study with improved diagnostic accuracy. Ann Emerg Med. 1996. 28(1):10–17	89
80	Murphy AA, Kettel LM, Nager CW, et al.: Operative laparoscopy versus laparotomy for the management of ectopic pregnancy—a prospective trial. Fertil Steril. 1992. 57(6):1180–1185	89
81	Sherman D, Langer R, Sadovsky G, et al.: Improved fertility following ectopic pregnancy. Fertil Steril. 1982. 37(4):497–502	89
82	Schumacher A, Brachwitz N, Sohr S, et al.: Human chorionic gonadotropin attracts regulatory T cells into the fetal-maternal interface during early human pregnancy. J Immunol. 2009. 182(9):5488–5497	88
83	Mol Bwj, Lijmer JG, Ankum WM, et al.: The accuracy of single serum progesterone measurement in the diagnosis of ectopic pregnancy: a meta-analysis. Hum Reprod. 1998. 13(11):3220–3227	88
84	Zhang J, Thomas AG, Leybovich E: Vaginal douching and adverse health effects: a meta-analysis. Am J Public Health. 1997. 87(7):1207–1211	86
85	VanVoorhis WC, Barret LK, Sweeney YTC, et al.: Repeated *Chlamydia trachomatis* infection of *Macaca nemestrina* fallopian tubes produces a Th1-like cytokine response associated with fibrosis and scarring. Infect Immun. 1997. 65(6):2175–2182	86
86	Cheginl N, Flanders KC: Presence of transforming growth-factor-beta and their selective cellular-localization in human ovarian tissue of various reproductive stages. Endocrinology. 1992. 130(3):1707–1715	86
87	Yovich JL, Turner SR, Murphy AJ: Embryo transfer technique as cause of ectopic pregnancies in invitro fertilization. Fertil Steril. 1985. 44(3):318–321	86
88	Peterson HB, Xia ZS, Hughes JM, et al.: The risk of ectopic pregnancy after tubal sterilization. New Engl J Med. 1997. 336(11):762–767	85
89	Mage G, Pouly JL, Dejoliniere JB, et al.: A preoperative classification to predict the intrauterine and ectopic pregnancy rates after distal tubal microsurgery. Fertil Steril. 1986. 46(5):807–810	85
90	Fatum M, Rojansky N: Laparoscopic surgery during pregnancy. Obstet Gynecol Surv. 2001. 56(1):50–59	84
91	Fernandez H, Benifla JL, Lelaidier C, et al.: Methotrexate treatment of ectopic pregnancy—100 cases treated by primary transvaginal injection under sonographic control. Fertil Steril. 1993. 59(4):773–777	84
92	Nyberg DA, Mack LA, Jeffrey RB, et al.: Endovaginal sonographic evaluation of ectopic pregnancy—a prospective-study. Am J Roentgenol. 1987. 149(6):1181–1186	83
93	Decherney AH, Romero R, Naftolin, F: Surgical-management of unruptured ectopic pregnancy. Fertil Steril. 1981. 35(1):21–24	83
94	Bustillo M, Yee B: Assisted reproductive technology in the United States: 1996 results generated from the American Society for Reproductive Medicine/Society for Assisted Reproductive Technology Registry. Fertil Steril. 1999. 71(5):798–807	82
95	Lipscomb GH, Bran D, Mccord ML, et al.: Analysis of three hundred fifteen ectopic pregnancies treated with single-dose methotrexate. Am J Obstet Gynecol. 1998. 178(6):1354–1356	82
96	Condous G, Okaro E, Khalid A, et al.: The accuracy of transvaginal ultrasonography for the diagnosis of ectopic pregnancy prior to surgery. Hum Reprod. 2005. 20(5):1404–1409	81
97	Wang HB, Guo Y, Wang, DZ et al.: Aberrant cannabinoid signaling impairs oviductal transport of embryos. Nat Med. 2004. 10(10):1074–1080	81
98	Zeitz J: Assisted reproductive technology in the United States and Canada: 1995 results generated from the American Society for Reproductive Medicine Society for Assisted Reproductive Technology Registry. Fertil Steril. 1998. 69(3):389–398	81
99	Mccord ML, Muram D, Buster, JE et al.: Single serum progesterone as a screen for ectopic pregnancy: exchanging specificity and sensitivity to obtain optimal test performance. Fertil Steril. 1996. 66(4):513–516	81
100	Kurman RJ: The morphology, biology, and pathology of intermediate trophoblast—a look back to the present. Hum Pathol. 1991. 22(9):847–855	81

**Fig. 2:**
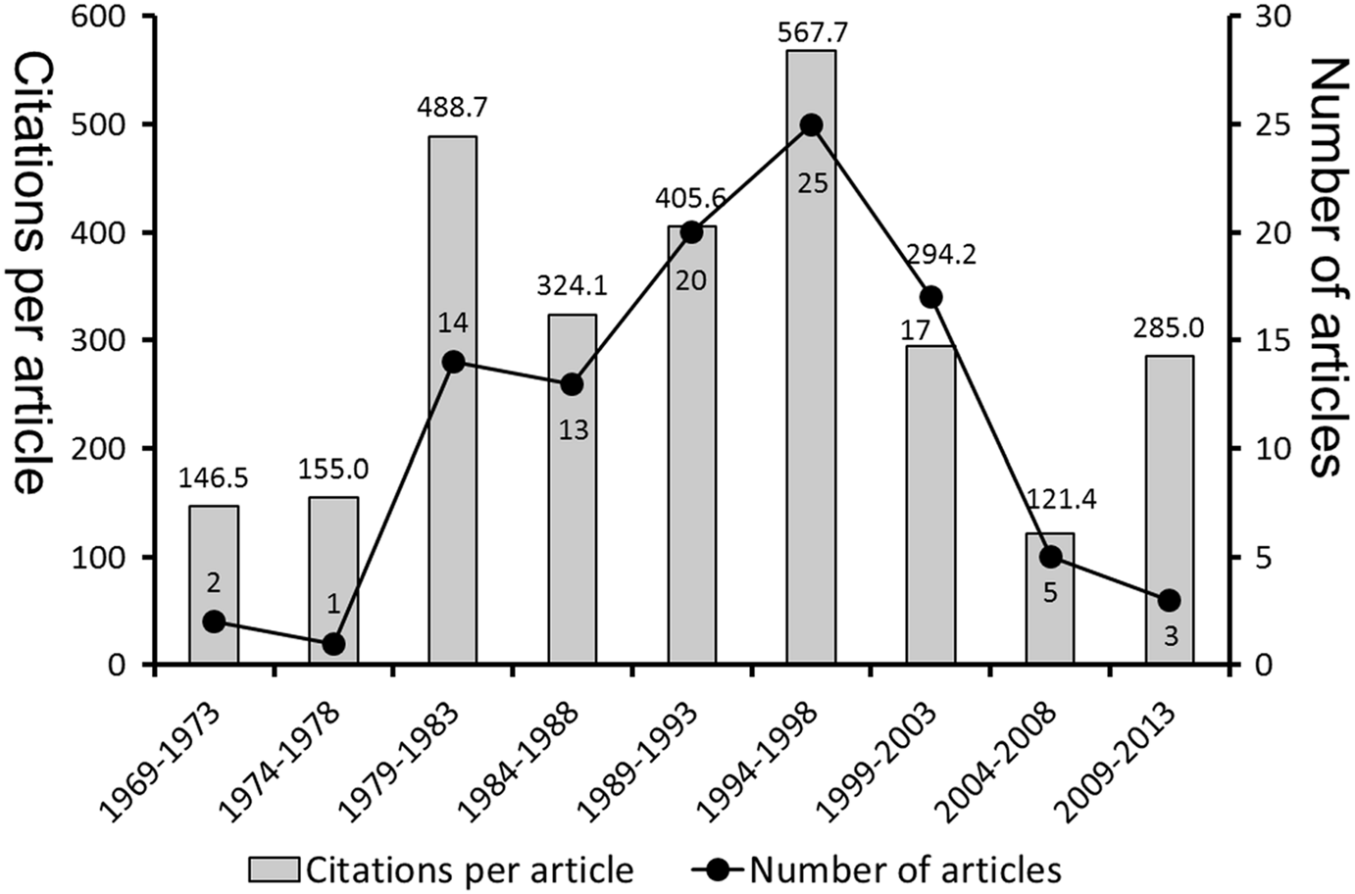
Flowchart of the number of the 100 top-cited papers in ectopic pregnancy per year

The most cited paper was contributed by Kalman et al. (1999) with 482 citations, whereas the 100th paper by Chegini and Flanders (1992) was cited 86 times. The mean number of citations was 132 (SD 69.76). The oldest citation classic on the list was ranked at position 62 and was published in 1969 by Kobayashi et al. (1969) (96 citations), and the most recent paper was at position 82 and was published in 2009 (88 citations) by Schumacher et al. (2009). In analyzing the list by publishing decade, most of the top 100 papers were published in the 1990s, and no top 100 paper was published in the 2010s (Fig. 2).

**Fig. 3:**
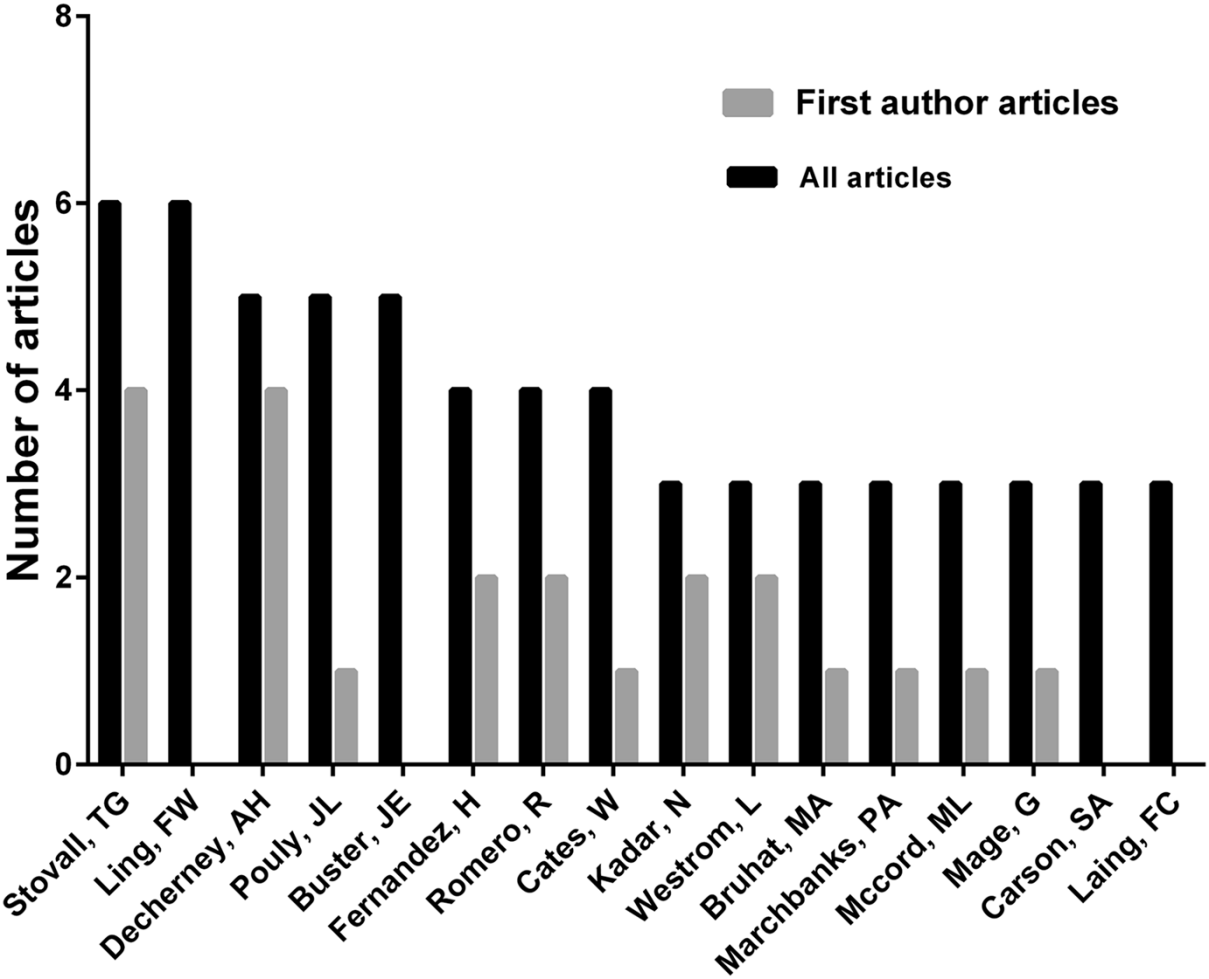
The top-ranked authors who published the 100 top-cited papers in ectopic pregnancy

Several authors published multiple papers in the top 100 list (Fig. 3; Table 2). Both Stovall TG and Ling FW, who published six papers, appeared at the top of the list, followed by Decherney AH, Pouly JL and Buster JE with five papers. In regard to the first author and corresponding author, Stovall TG appeared at the top of the list with four papers, followed by Decherney AH also with four first author papers, in which Barnhart was the corresponding author as well.

**Table 2:** List of authors who published more than 3 of most 100 cited papers in ectopic pregnancy

Rank	Author	No. of articles	First author	Corresponding author
1	Stovall TG	6	4	4
2	Ling FW	6	0	0
3	Decherney AH	5	4	4
4	Pouly JL	5	1	0
5	Buster JE	5	0	0
6	Fernandez H	4	2	1
7	Romero R	4	2	1
8	Cates W	4	1	1
9	Kadar N	3	2	2
10	Westrom L	3	2	2
11	Bruhat MA	3	1	3
12	Marchbanks PA	3	1	1
13	Mccord ML	3	1	1
14	Mage G	3	1	0
15	Carson SA	3	0	0
16	Laing FC	3	0	0

The first authors with more articles took precedence and then the corresponding author and at last the initials according to the order of the alphabet in the situation of equal numbers of articles

The journal of Fertility and Sterility published the highest number of papers (23), followed closely by Obstetrics and Gynecology (15), American Journal of Obstetrics and Gynecology (10), New England Journal of Medicine (6) and JAMA—Journal of The American Medical Association (6). The detailed results are shown in Table 3.

**Table 3:** List of journals that published more than 2 of top 100 cited articles in ectopic pregnancy

Rank	Journal	No. of articles	Total citation	Impact factor
1	Fertil Steril	23	2601	4.59
2	Obstet Gynecol	15	1937	5.175
3	Am J Obstet Gynecol	10	1700	4.704
4	JAMA-J Am Med Assoc	6	863	35.289
5	New Engl J Med	6	790	55.873
6	Hum Reprod	5	514	4.569
7	Radiology	4	377	6.867
8	Brit Med J	3	666	17.445
9	Sex Transm Dis	2	507	2.842
10	Ultrasound Obst Gyn	2	325	3.853
11	Brit J Obstet Gynaec	2	284	3.448
12	Am J Public Health	2	178	4.552

The journals with more total citation took precedence in the situation of equal numbers of articles

Of the top 100 articles, 65 papers were from the United States, followed by the United Kingdom and France (Table 4). In regard to institution contribution (Table 5), Yale University in Connecticut contributed seven papers, whereas the Center for Disease Control and Prevention in Atlanta, Georgia published six papers each. Of all of the articles, 36 resulted from multi-institutional collaboration, 4 from multinational collaboration, and 64 from individual institutions.

**Table 4:** List of countries that published the 100 top-cited articles in ectopic pregnancy

Rank	Countries	TP	TC	SP	CP
1	USA	65	8524	63	2
2	France	9	1196	9	0
3	UK	8	0	6	2
4	Canada	4	463	3	1
5	Sweden	4	420	2	2
6	Denmark	2	430	2	0
7	Finland	2	261	2	0
8	Netherlands	2	230	2	0
9	Israel	2	173	2	0
10	Belgium	2	111	1	1
11	Taiwan	1	143	1	0
12	Japan	1	96	1	0
13	Germany	1	88	1	0
14	Australia	1	0	0	1

TP refers to all the co-authors originating from the same country or territory contributed to the number of the 100 most cited articles in EP; TC refers to the number of 100 top-cited articles published by the first author originating from each country or territory; SP refers to single country or territory articles; CP refers to internationally collaborative articles. Rank: according to the order of TP, TC, SP, CP and the order of the alphabet of the initials

**Table 5:** List of institutions that published 2 or more of the 100 top-cited articles in ectopic pregnancy

Rank	Institutions	TP	TC	SP	CP
1	Yale Univ	7	972	7	0
2	Ctr Dis Control	6	702	2	4
3	Ctr Dis Control & Prevent	6	406	1	5
4	Univ Tennessee	5	667	5	0
5	Univ Calif San Francisco	4	268	2	2
6	Hop Antoine Beclere	4	187	1	3
7	Emory Univ	4	167	0	4
8	Vanderbilt Univ	3	267	2	1
9	Univ Washington	3	209	1	2
10	Univ Penn	3	196	2	1
11	Hop Bicetre	3	98	0	3
12	Univ Calif Los Angeles	2	266	1	1
13	Univ Helsinki	2	261	2	0
14	Univ Amsterdam	2	230	2	0
15	Univ Lund Hosp	2	219	1	1
16	Harvard Univ	2	194	1	1
17	Univ Vermont	2	185	2	0
18	Baylor Coll Med	2	176	2	0
19	Amer Soc Reprod Med	2	163	2	0
20	Univ So Calif	2	90	1	1
21	Aarhus Univ	2	0	0	2
22	Univ Alabama	2	0	0	2
23	Univ Michigan	2	0	0	2

TP refers to all the co-authors originating from the same institution contributed to the number of the 100 most cited articles in EP; TC refers to the citation times of the 100 most cited articles in EP published as the first author institution; SP refers to single institution articles; CP refers to inter-institutionally collaborative articles. Rank: according to the order of TP, TC, SP, CP and the order of the alphabet of the initials

Additionally, high-frequency keywords that appeared more than ten times in the 100 most cited articles, including EP, treatment, risk factors, methotrexate, diagnosis, tubal pregnancy, *Chlamydia trachomatis* infections, human chorionic gonadotropin (HCG), pelvic inflammatory disease, ultrasound, were screened to determine the hot topics in EP research (Table 6).

**Table 6:** The categories of research hotspots in 100 most cited articles in ectopic pregnancy

Rank	Key word	Frequency
1	Ectopic pregnancy	79
2	Treatment	26
3	Risk factors	17
4	Methotrexate	16
5	Diagnosis	15
6	Tubal pregnancy	14
7	*Chlamydia trachomatis* infections	13
8	HCG (Human Chorionic-Gonadotropin)	12
9	Pelvic inflammatory disease	12
10	Ultrasound	11

## Discussion

The term “citation classics” was first introduced by Eugene Garfield in 1987 in a study to identify the 100 most-cited JAMA articles (Garfield 1987). Since then, citation classics have been studied by many authors in various fields. The number of times that an article is cited is a good way of measuring the impact that the article has on a specific field or topic and, in turn, allows both the author and the journal to be evaluated (Garfield 1972). In our study, the SCI-expanded was used to identify the 100 most cited papers in EP to produce a list of citation classics in this field. This list provides us with a source of great value in terms of the authors and topics that have had a profound influence in the area of EP over the past 50 years.

The present study summarizes several features of influential articles in EP research over the past 50 years. For instance, we found that half of the 100 most cited papers were published in the 1990s, which suggests that older papers are cited more (Picknett and Davis 1999). The articles in 1990s were neither too old to have some outdated opinions nor too early to have time to be proven or cited. In fact, it has been found that the true impact and importance of an article cannot often be precisely assessed for at least two decades after it is published (Baltussen and Kindler 2004).

A high citation frequency also demonstrates that other authors have formulated opinions on the topic and that it has generated discussion and debate (Nason et al. 2013). In our study, these top 100 articles were published in 32 peer-reviewed journals of high quality, as demonstrated by their impact factor (mean 10.421, range 1.535–55.873). The impact factor of a journal is generally accepted as a representation of the scientific quality of a publication. With further analysis, we found that most of the top-cited articles were published in high-impact journals, which is consistent with the results of other reviews. It is generally presumed that articles that are published in high-impact journals are more likely to have an extensive popularity among readers and thus have a greater potential for citation, which in turn maintains the high impact factor of these journals. This factor also supports the well-known paradigm that top-cited articles are often published in journals topping the impact factor list, which in turn maintains the high impact factor of these journals (Garfield 2006). Additionally, it was found that American authors tended to cite local papers (Campbell 1990), and that US reviewers had a significant preference to accept papers written by native researchers (Link 1998).

Additionally, we found that all of the papers in the top 100 were written in the English language and that a majority of them were from the United States. One of the underlying reasons might be due to the large population of senior researchers, adequate research budgets and superior scientific research conditions for scientific investigation. Besides, because of the powerful influence of English-speaking countries like USA, UK and so on and the fact that English is the official language using by most countries and is the world’s most extensive second language, English is widely used all over the word. Collaboration has increased at the author, institution and country levels, which is supported by many earlier studies (Kliegl and Bates 2011). Of the top 100 cited papers, 64 came from individual institutions, 36 came from multi-institutional collaboration, and 4 came from multinational collaboration. This finding reflects that teamwork awareness, in some cases, is of great importance and that scientific collaboration plays an indispensable role in the progress of scientific research.

EP has been defined as pregnancy that develops after implantation of the blastocyst anywhere other than the endometrium lining of the uterine cavity. It remains a major gynecological problem in contemporary gynecological practice and continues to be an important cause of morbidity and mortality in women. Therefore, many studies have targeted the pathogenesis, diagnosis or treatment application to improve future prognosis. According to the implantation site of the blastocyst, EP is divided into tubal pregnancy, ovarian pregnancy, abdominal pregnancy, or intraligamentary pregnancy. Among the sites of EP, more than 95 % of EPs occur in the fallopian tubes. Some special-site EPs such as cesarean scar pregnancy (Godin et al. 1997; Hemminki and Merilainen 1996; Jurkovic et al. 2003; Seow et al. 2004; Sorbi et al. 2013), interstitial pregnancy (Lau and Tulandi 1999), cornual pregnancy (Timortritsch et al. 1992), and cervical pregnancy (Frates et al. 1994) were discussed in the 100 most cited papers.

The risks of an EP vary across women. Among the 100 articles, more than a quarter of them emphasized the risk factors of EP, among which the relationship between *Chlamydia trachomatis* infection and EP was discussed mostly (Brunham et al. 1986; Cates and Wasserheit 1991; Chow et al. 1990; Egger et al. 1998; Hillis et al. 1997; VanVoorhis et al. 1997). Additionally, the authors focused on other risk factors that can lead to EP, such as pelvic inflammatory disease (PID), smoking, in vitro fertilization (IVF), the use of intrauterine contraceptive devices (IUD), and vaginal douching (Aral et al. 1992; Castles et al. 1999; Hillis et al. 1993; Logerotlebrun et al. 1995; Ory 1981; Rogers 2009; Westrom et al. 1992; Zhang et al. 1997). The phenomenon reminds us that the etiology and pathogenesis of EP for clinical and basic research has attracted close attention from many senior researchers.

The diagnosis of EP has been a hot topic in research across the world. A timely, early diagnosis can help patients obtain better pregnancy outcomes. Ultrasonography and β-hCG levels are important in the early diagnosis of EP (Crochet et al. 2013). Moreover, the combined application of ultrasound and β-hCG levels has great value in a precise diagnosis (Cacciatore et al. 1990; Kadar et al. 1981). We also found that the articles relating to early diagnosis were mainly cited before the 1990s, indicating that the effect of early diagnosis of EP on clinical practice and basic research still needs to be explored.

The treatment of EP has drawn attention from modern researchers. The treatments presented in the classic articles include expectant management and medical and surgical protocols. The focus of treatment is to select a safer and more effective method to preserve reproductive potential. Methotrexate treatment, especially single-dose methotrexate, is thought to reduce the potential cost and morbidity of hospitalization and surgery, which is discussed mostly as well (Glock et al. 1994; Stovall and Ling 1993; Stovall et al. 1991). Following EP, fertility is another topic that is commonly discussed in these classic articles (Sherman et al. 1982) which show improved intrauterine pregnancy after an ectopic pregnancy. Besides, ESEP study and DEMETER study show high rate of 2 years intrauterine pregnancy after an ectopic pregnancy which match the conclusion (Fernandez et al. 2013; Mol et al. 2014).

Although the top 100 articles have proved to be most useful to the vastly larger population of practicing scientists, some limitations are existed in our study (Van Noorden et al. 2014). First, the top-cited articles were always the older papers because of the limited life span of literature. Therefore, some points need to be updated, and such updates are likely to identify trends in research patterns (Garfield 1972). Another important problem with this type of analysis is the “obliteration by incorporation” phenomenon (Garfield 1987). This issue describes the process in which information from truly classic papers becomes cited less frequently and is absorbed into the body of current knowledge (Kelly et al. 2010). Second, the words that we used as subject terms were only “ectopic pregnancy” and “ectopic pregnancies”, which may miss some citations related to our analysis such as those indexed with extrauterine pregnancy or heterotopic pregnancy. Furthermore, the only database that we searched was the SCI-expanded, and those articles published before 1965 were excluded from our study. Therefore, some “classic” articles from other databases or before 1965 may have also been missed in this analysis. Additionally, self-citation, journal bias and language bias were not controlled for in our study, and these issues may have affected our research, whereas citation analysis is still a feasible tool to comprehensively recognize the advances of EP research in the past and future research.

## Conclusions

Bibliometric analysis was used to provide a historical perspective on the progress in EP research over the past 50 years. The citation increases as time goes by, and it reaches its peak in the 1990s. Articles originating from the United States and published in high-impact journals were most likely to be cited in the field of EP research. The risk factors of EP like *Chlamydia trachomatis* infections and the treatment of EP especially like methotrexate medical management were screened to present the hotspots of EP research.
